# Changes in pregnancy-related serum biomarkers early in gestation are associated with later development of preeclampsia

**DOI:** 10.1371/journal.pone.0230000

**Published:** 2020-03-03

**Authors:** Shiying Hao, Jin You, Lin Chen, Hui Zhao, Yujuan Huang, Le Zheng, Lu Tian, Ivana Maric, Xin Liu, Tian Li, Ylayaly K. Bianco, Virginia D. Winn, Nima Aghaeepour, Brice Gaudilliere, Martin S. Angst, Xin Zhou, Yu-Ming Li, Lihong Mo, Ronald J. Wong, Gary M. Shaw, David K. Stevenson, Harvey J. Cohen, Doff B. Mcelhinney, Karl G. Sylvester, Xuefeng B. Ling

**Affiliations:** 1 Department of Cardiothoracic Surgery, Stanford University School of Medicine, Stanford, CA, United States of America; 2 Clinical and Translational Research Program, Betty Irene Moore Children's Heart Center, Lucile Packard Children’s Hospital, Palo Alto, CA, United States of America; 3 Department of Surgery, Stanford University School of Medicine, Stanford, CA, United States of America; 4 Department of Pediatrics, Stanford University School of Medicine, Stanford, CA, United States of America; 5 Department of Emergency, Shanghai Children’s Hospital, Shanghai Jiao Tong University School of Medicine, Shanghai, China; 6 Department of Health Research and Policy, Stanford University, Stanford, CA, United States of America; 7 Department of Obstetrics and Gynecology, Stanford University School of Medicine, Stanford, CA, United States of America; 8 Department of Anesthesiology, Perioperative, and Pain Medicine, Stanford University School of Medicine, Stanford, CA, United States of America; 9 Tianjin Key Laboratory of Cardiovascular Remodeling and Target Organ Injury, Pingjin Hospital Heart Center, Tianjin, China; 10 Department of Obstetrics and Gynecology, University of California San Francisco-Fresno, Fresno, CA, United States of America; University of Mississippi Medical Center, UNITED STATES

## Abstract

**Background:**

Placental protein expression plays a crucial role during pregnancy. We hypothesized that: (1) circulating levels of pregnancy-associated, placenta-related proteins throughout gestation reflect the temporal progression of the uncomplicated, full-term pregnancy, and can effectively estimate gestational ages (GAs); and (2) preeclampsia (PE) is associated with disruptions in these protein levels early in gestation; and can identify impending PE. We also compared gestational profiles of proteins in the human and mouse, using pregnant heme oxygenase-1 (HO-1) heterozygote (Het) mice, a mouse model reflecting PE-like symptoms.

**Methods:**

Serum levels of placenta-related proteins–leptin (LEP), chorionic somatomammotropin hormone like 1 (CSHL1), elabela (ELA), activin A, soluble fms-like tyrosine kinase 1 (sFlt-1), and placental growth factor (PlGF)–were quantified by ELISA in blood serially collected throughout human pregnancies (20 normal subjects with 66 samples, and 20 subjects who developed PE with 61 samples). Multivariate analysis was performed to estimate the GA in normal pregnancy. Mean-squared errors of GA estimations were used to identify impending PE. The human protein profiles were then compared with those in the pregnant HO-1 Het mice.

**Results:**

An elastic net-based gestational dating model was developed (R^2^ = 0.76) and validated (R^2^ = 0.61) using serum levels of the 6 proteins measured at various GAs from women with normal uncomplicated pregnancies. In women who developed PE, the model was not (R^2^ = -0.17) associated with GA. Deviations from the model estimations were observed in women who developed PE (*P* = 0.01). The model developed with 5 proteins (ELA excluded) performed similarly from sera from normal human (R^2^ = 0.68) and WT mouse (R^2^ = 0.85) pregnancies. Disruptions of this model were observed in both human PE-associated (R^2^ = 0.27) and mouse HO-1 Het (R^2^ = 0.30) pregnancies. LEP outperformed sFlt-1 and PlGF in differentiating impending PE at early human and late mouse GAs.

**Conclusions:**

Serum placenta-related protein profiles are temporally regulated throughout normal pregnancies and significantly disrupted in women who develop PE. LEP changes earlier than the well-established biomarkers (sFlt-1 and PlGF). There may be evidence of a causative action of HO-1 deficiency in LEP upregulation in a PE-like murine model.

## Introduction

Placental protein expression plays a crucial biological role during normal pregnancies. The normal progression of a human pregnancy is associated with a precisely-timed regulation of the expression of maternal and placental proteins [1, 2]. Similarly, the placenta, an endocrine gland unique to pregnancy, secretes hormones that fluctuate with respect to the gestational week of pregnancy. However, these hormones have not been useful in the development of molecular metrics to estimate gestational age (GA) or phenotyping complicated pregnancies prior to overt clinical manifestations of specific pathologic states like preeclampsia (PE) [3, 4].

PE, a pregnancy-related placental vascular disorder affecting 5–8% of all pregnancies [5, 6], is thought to be a multisystem disorder of pregnancy driven by alterations in placental function and resolved by the delivery of the placenta and fetus [7]. Some pregnancy-associated, placenta-related markers have been observed to display different profiles in normal pregnancies compared with pregnancies with PE. Chorionic somatomammotropin hormone like 1 (CSHL1; also called human placental lactogen) is selectively expressed in placental villi with an important role in regulating placental growth. Leptin (LEP) has been suggested to be involved in placental and fetal growth [8]. The relationship between LEP and PE has been demonstrated in a number of studies [9–17]. Circulating levels of activin A, a member of the tumor growth factor protein family, can increase as early as 10–15 weeks of pregnancy in women who subsequently develop PE [18]. Elevated placental levels of angiogenic factors (soluble fms-like tyrosine kinase or sFlt-1) and decreased levels of anti-angiogenic factors (placental growth factor, PlGF) have also been implicated in the pathogenesis of PE [19–25]. As such, the sFlt-1/PlGF ratio has been proposed as an index to identify women with PE [26, 27]. Significant increases in sFlt-1 levels were also observed in sera of pregnant heme oxygenase (HO)-1 heterozygote (Het, HO-1^+/-^) mice, where the deficiency in HO-1 results in PE-like symptoms [28]. Recent work by Ho et al showed that, in mice, PE is associated with a deficiency in elabela (ELA), a placental hormone that enhances human trophoblast invasiveness *in vitro* [29].

In this study, we targeted LEP, CSHL1, ELA, activin A, sFlt-1, and PlGF as biomarker candidates for estimating GA and identifying an impending onset of PE. These 6 proteins are not only associated with the placenta and reflect placental growth [8, 30–32]; but also because we found that CSHL1 and PlGF were highly correlated to GA from 10 to 30 weeks of pregnancy by our longitudinal quantitative analysis [2] and that the levels of these proteins differ in women with PE compared with women with normal pregnancies. The predictive value of sFlt-1 and PlGF in PE has been validated by a number of studies [19–27]. Elevations of LEP, CSHL1, and activin A levels were observed early in gestation in women who subsequently develop PE [33–35]. ELA deficiency is associated with PE-like symptoms in mice [29].

We hypothesized that serum levels of placenta-related proteins, LEP, CSHL1, ELA, activin A, sFlt-1, and PlGF are temporally regulated over the course of pregnancy, and profiles of their circulating levels may collectively reflect the normal progression of a term pregnancy. We further hypothesized that disruptions of these profiles in early gestation are associated with placental abnormalities and signal an increased risk of developing PE. We sought to model the longitudinal changes in serum levels of these protein to estimate GA. In addition, we explored whether temporal disruptions in these profiles early in gestation are harbingers of placental pathology and the development of subsequent PE. The model was first developed in human sera and then tested in both human and mouse sera. We chose the HO-1 Het mouse model because during pregnancy these mice display PE-like symptoms, such as elevated diastolic blood pressures and increases in plasma sFlt-1 levels [28]. Furthermore, unlike other mouse models of PE [36, 37], the placentas of these mice have vascular defects, which mimic early-onset PE [28, 38].

## Materials and methods

### Study design

The study was conducted in three phases: (1) using ELISA methods to characterize the normal pattern of serum placenta-related protein levels; (2) modeling a protein-based GA estimation of normal pregnancies and identifying deviations; and (3) exploration of the protein-based GA estimation with a mouse PE model.

### Patients and blood collection

Subjects were selected retrospectively from a cohort of pregnant women who were invited to participate between November 2012 and May 2016 in a prospective longitudinal study sponsored by the March of Dimes. Approval was obtained from the Stanford University Institutional Review Board. Women were eligible for enrollment if they were at least 18 years of age and at their first trimester of a singleton pregnancy. After written informed consent was obtained, blood was collected up to 4 time points, representing the first, second, and third trimesters of pregnancy and 6-weeks postpartum. From this cohort, we analyzed sera from women who had normal uncomplicated pregnancies or who later received a diagnosis of PE. Blood was collected at 1 to 3 time-points prior to a confirmatory diagnosis of PE. GAs were determined by ultrasound measurement.

The diagnosis of PE was made according to the American College of Obstetricians and Gynecologists criteria [39], as follows: a persistent systolic blood pressure ≥ 140 mmHg, or a diastolic blood pressure ≥ 90 mmHg after 20 wks’ GA in a woman with previously normal range of blood pressures in conjunction with one or more of the following: new-onset proteinuria, new-onset thrombocytopenia, impaired liver function, renal insufficiency, pulmonary edema, or visual or cerebral disturbances in the absence of proteinuria. The PE subgroup in this study included enrolled women who fulfilled the PE diagnostic criteria. The normal subgroup included women who had full-term deliveries and without any complications of pregnancy, or any history of preterm birth or PE.

### Animal model study

For the mouse studies, approval was obtained from the Institutional Animal Care and Use Committee at Stanford University. Wild-type (WT) FVB/N male and female mice were purchased from Charles River Laboratories (Wilmington, MA). C57BL/6 HO-1/KO mice were backcrossed with FVB mice to produce an FVB HO-1/KO mouse line as previously described [38]. Mice were mated at 6–10 wks of age. All animals were allowed food and water ad libitum and maintained according to institutional guidelines of Stanford University. Gestational ages were calculated by the presence of a vaginal plug and deemed as E0.5. Mouse line maintenance and genotyping were performed as previously described [28]. Blood was serially collected by submandibular puncture. No anesthesia was used. Mice were then transferred to microtainer tubes containing EDTA. Tubes were then spun at 13,000 x g for 1 minute and then transferred to 1.5 mL microfuge tubes and then stored at -80°C until analysis. Sera were collected from pregnant HO-1 Het or WT dams at 1 to 3 time-points between E7.5 to E18.5.

### ELISAs

Serum placental proteins from pregnant women or mice were measured using species-specific commercial kits: LEP (R&D System Inc., MN, USA); CSHL1 (Mybiosource, San Diego, CA, USA); ELA (Peninsula Laboratories International, Inc., San Carlos, CA, USA); activin A (R&D System Inc.); sFlt-1 (R&D System Inc.,); and PlGF (R&D System Inc.).

### Statistical analyses

Patient demographic data were analyzed using the “Epidemiological Calculator” (R epicalc package). Hypothesis testing was performed using Mann-Whitney U-tests (two-tailed). Samples collected ≥ 30 weeks of gestation or having any of the placenta-related protein measurements out of limits on the standard curves were excluded from the cohort for modeling. An elastic net (EN) algorithm [40] was applied for estimating GAs using the ELISA data. EN is a statistical learning technique that has been used for GA estimation [2, 41], biomarker discovery [42, 43], and risk prediction [44]. The input of the model was the ELISA data of each analyte, and the output was the GA at sample collection. The model was trained and validated with the women with normal pregnancies, and then tested in women who developed PE. The detail of the modeling procedure is shown in S1 File.

The mean squared error (MSE) of the GA model was used to separate PE patients from women with normal pregnancies. One MSE was calculated for each woman by comparing the observed GA with the model-predicted GA over the associated longitudinal samples. MSE values of normal women and PE women were compared, and receiver operating characteristic (ROC) curves and Mann-Whitney U-tests were calculated to test the performance of MSE in classifying women.

The EN model was then adjusted using 5 analytes as inputs (ELA was excluded see below). The model performance was assessed by R^2^. The role of each analyte in differentiating complicated from normal pregnancies was explored by analyzing the distribution of the concentrations at different GAs. Comparisons were made between the human and mouse to identify the common behaviors in proteins that were associated with the outcome of PE. Loess regression, Mann-Whitney U-tests, and fold changes were used for the analyses.

## Results

### Samples

**Fig 1:**
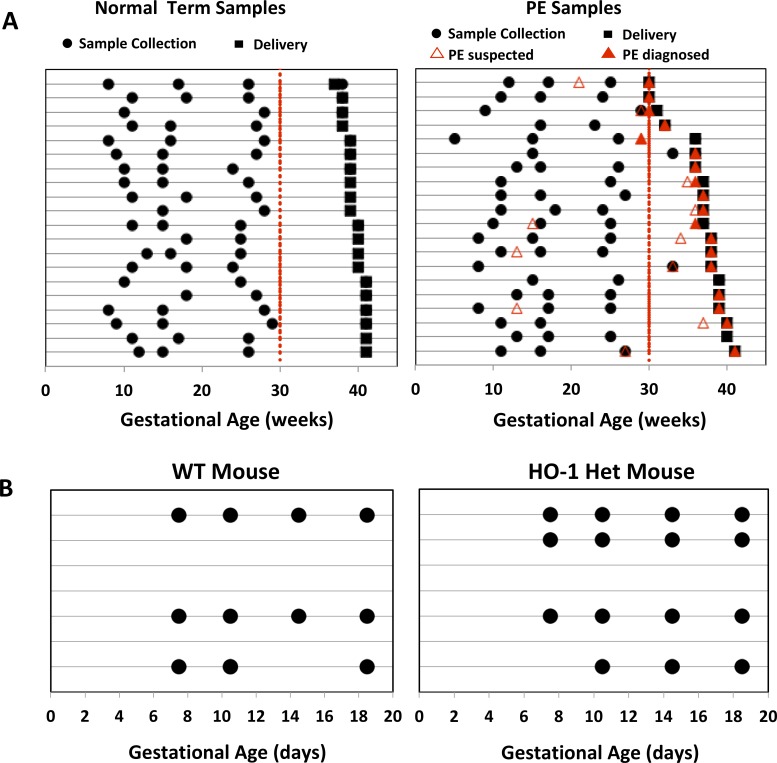
(A) Serial blood sampling from each normal term and PE subject at different GAs. Times of sample collections, infant deliveries, suspected PE, and confirmatory PE diagnoses of individual women (denoted by each row) are represented by black circles, black squares, red unfilled triangles, and red-filled triangles, respectively. (B) Serial blood collection from each pregnant WT (left) and HO-1 Het (right) mouse at different GAs. Sample collection days and individual mice are represented by filled circles and lines, respectively.

The demographics of the forty pregnant women (20 term pregnancies, 20 with PE) in our study cohort are listed in Table 1. Sample collection times for each woman are shown on Fig 1A. 10 women (4 normal pregnancies, 6 with PE) were excluded from the EN-based modeling because samples were either not collected before 30 weeks of gestation or had at least 1 protein candidate that was out of limits on its standard curve. The latter was done because outliers on the standard curve might cause distortion of our continuous regression analysis. A total of 30 women (16 normal pregnancies, 14 with PE) were therefore used for these analyses. Our training cohort included 10 patients who delivered at term (≥ 37 weeks’ GA). An independent cohort of 6 women who delivered at term and 14 women diagnosed with PE were subsequently enrolled for the validation study of normal pregnancy and then tested on women who developed PE.

**Table 1:** Subject demographics.

Characteristic	Overall Normal (n = 20)	PE (n = 20)
**Race, n (%)**		
**White**	20 (100)	9 (45)
**Asian**	0 (0)	5 (25)
**African-American**	0 (0)	1 (5)
**Other**	0 (0)	5 (25)
**Age, mean (SD), years**	31.9 (4.8)	31.8 (6.0)
**GA at delivery, mean (SD), weeks**	39.5 (1.2)	36.7 (3.3)
**Early-onset PE (Diagnosed < 34 weeks’ GA), n (%)**	NA	5 (25)
**Diagnosed with severe PE, n (%)**	NA	10 (50)

The approach was also tested with serum samples collected longitudinally from pregnant WT (n = 3 with 11 samples) and HO-1 Het (n = 4 with 15 samples) mice (Fig 1B). Blood samples were collected at E7.5, E10.5, E14.5, and E18.5.

### A placenta-related, protein-based GA estimation of human pregnancy

**Fig 2:**
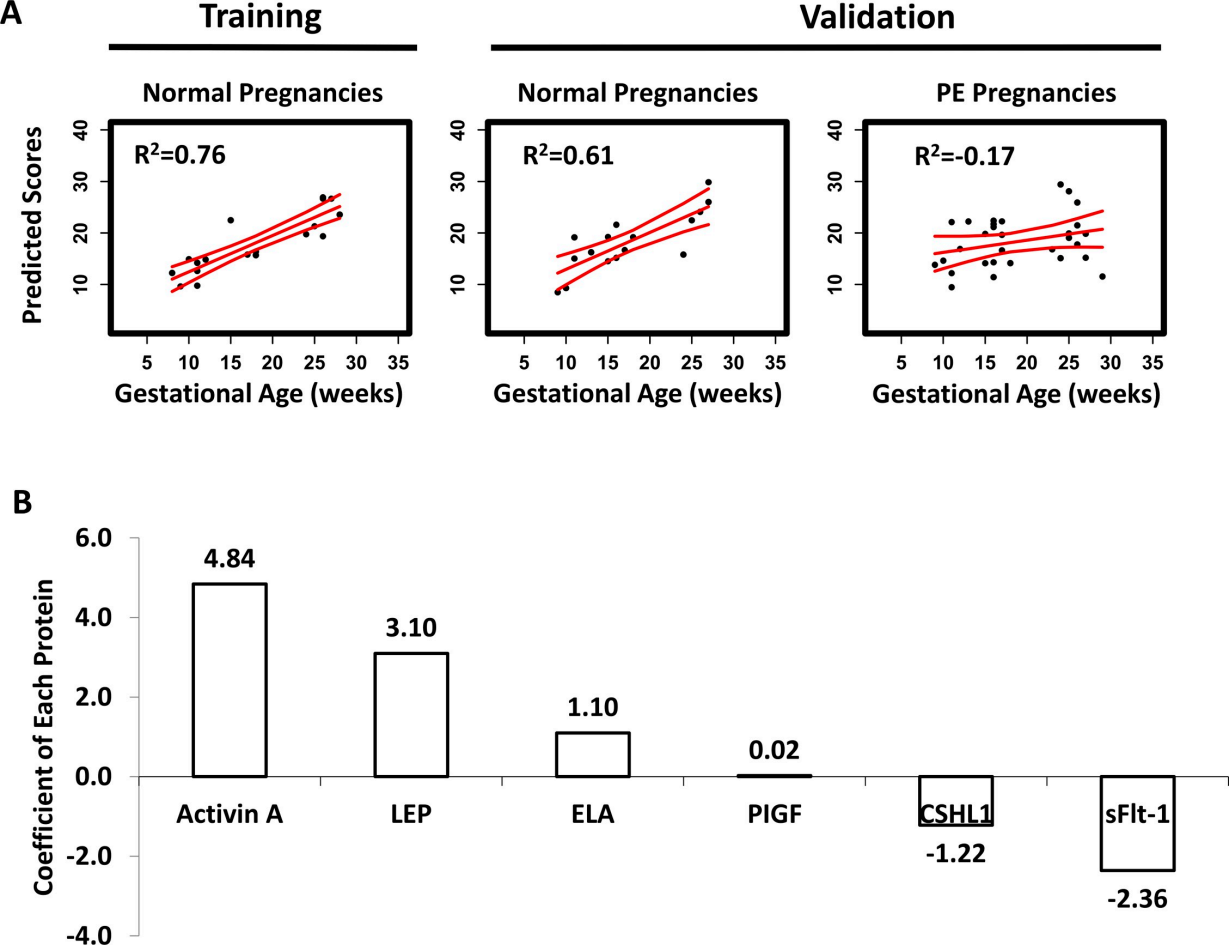
(A) The results of the EN model developed with serial sampling analyses of 6 placenta-related proteins, dating GAs in normal term pregnancies for both training and validation cohorts, and the results with the patients who developed PE. R^2^ was calculated as 1-RSS/TSS, where RSS is a residual sum of squares, and TSS is a total sum of squares. (B) Coefficients of each protein analyte in the EN model. Positive and negative values indicate positive and negative correlations, respectively, between GA and the serum protein concentrations. Predicted scores in (A) were calculated based on the coefficients of the 6 proteins shown in (B).

**Fig 3:**
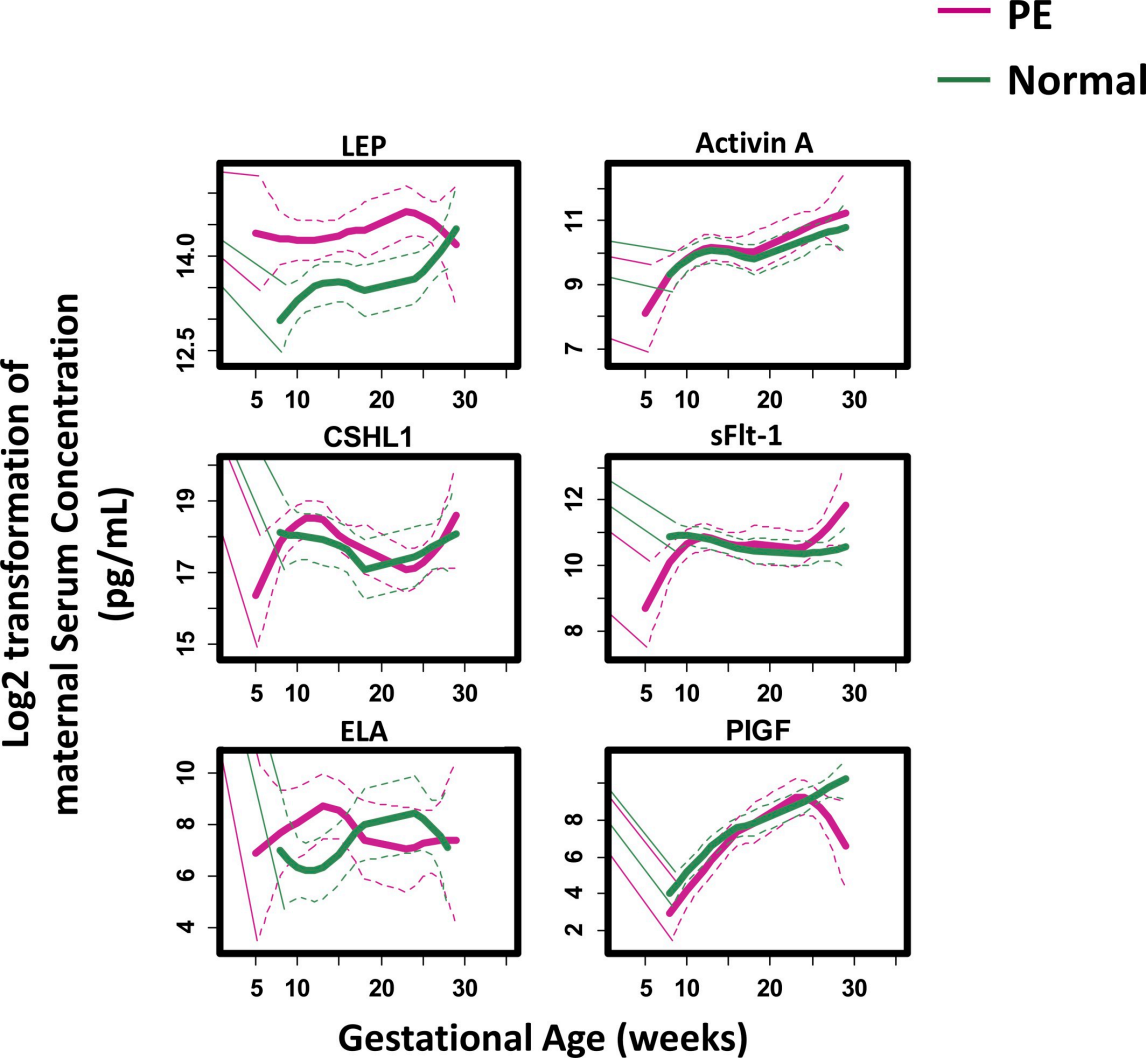
Maternal serum concentrations of the 6 studied placenta-related proteins plotted as a function of GA. Loess smooth function was applied to demonstrate the trend of the proteins in normal term (green line) and PE (red line) pregnancies, respectively. Color-matched dotted lines show the 95% confidence interval for each cohort.

We hypothesized that levels of circulating placenta-related proteins throughout pregnancy reflect the temporal progression of a normal human term pregnancy, and thus can effectively estimate GA. Using an EN algorithm, we developed a 6-protein model using a training cohort of 10 pregnant women that was strongly associated with GA at the time of sampling (R^2^ = 0.76, *P* = 2x10^-7^, Fig 2A, left panel). The EN model was prospectively tested using sera serially collected from 6 additional normal, full-term pregnant women. The EN model was found to predict GA at time of sampling in this independent normal cohort (R^2^ = 0.61, *P* = 2x10^-4^, Fig 2A, middle panel). EN model coefficients of each protein in the model are shown in Fig 2B. Together, the analyses confirmed that there is a highly-regulated temporal pattern of expression of these 6 proteins in sera over the course of pregnancy (Fig 3).

### The placenta-related, protein-based GA estimation malfunctions in PE

Based on the above findings, we hypothesized that our EN model can identify abnormal phenotypes, such as in PE, that may have an attendant disrupted placenta-related protein profile. In contrast to the normal cohort (training R^2^ *=* 0.76 and testing R^2^ = 0.61, Fig 2A), the EN model did not predict GA at time of sampling and yielded random data predictions in the PE cohort (Fig 2A, right panel, R^2^ = -0.17, *P* = 0.2). These findings suggest that the protein-based GA estimation (and profile) is disrupted in PE.

The pathogenesis of PE is complex and progresses from an asymptomatic stage, characterized by undetected placental abnormalities during the first trimester to a symptomatic stage with proteinuria and hypertension in late gestation [45]. Our analyses revealed unique longitudinal expression patterns of serum protein levels of specific biomarkers (Fig 3). Specifically, LEP, CSHL1, and ELA levels at approximately 10 weeks of gestation differentiated women with PE from women with uncomplicated, full-term pregnancies, indicating that the pathogenesis of PE may arise very early in gestation. In addition, differences in activin A levels begin to appear around 20 weeks of gestation and in sFlt-1 and PlGF after 25 weeks. Examination of the profiles of the levels of these proteins revealed significant gestational windows (5–9, 10–14, 15–25, 26–33, and 27–38 weeks’ GA, Table 2, S1 Fig, and S1 Table) specific for each biomarker. These findings are in line with our longitudinal biomarker trending analyses (Fig 3). Since there was a positive association [8] between maternal serum LEP concentrations and body mass index (BMI) (and consequently, gestational weight gain) during pregnancy, we normalized serum LEP levels by BMI and found similar results (S2 Fig). Taken together, these data indicate that alterations in the profiles of serum levels of LEP, CSHL1, and ELA begin much earlier in GA than the changes in sFlt-1 (increase) and PlGF (decrease) at late GA.

**Table 2:** Comparisons of the serum levels of each protein between normal and PE pregnancies. Mann-Whitney U-test *P*-value was calculated. *0.005 < *P* < 0.05. **P < 0.005.

	5–9 weeks’ GA	10–14 weeks’ GA	15–25 weeks’ GA	26–33 weeks’ GA	27–38 weeks’ GA
**LEP**	0.02*	0.02*	3x10^-6^**	0.3	0.5
**CSHL1**	0.4	0.01*	0.3	0.7	0.9
**ELA**	0.9	0.03*	0.9	0.8	0.4
**Activin A**	0.5	0.5	0.8	0.04*	0.2
**sFlt-1**	0.2	0.3	0.8	0.02*	3x10^-3^**
**PlGF**	0.6	0.9	0.5	0.3	0.01*

### Disruption of the protein-based GA estimation identifies impending PE

**Fig 4:**
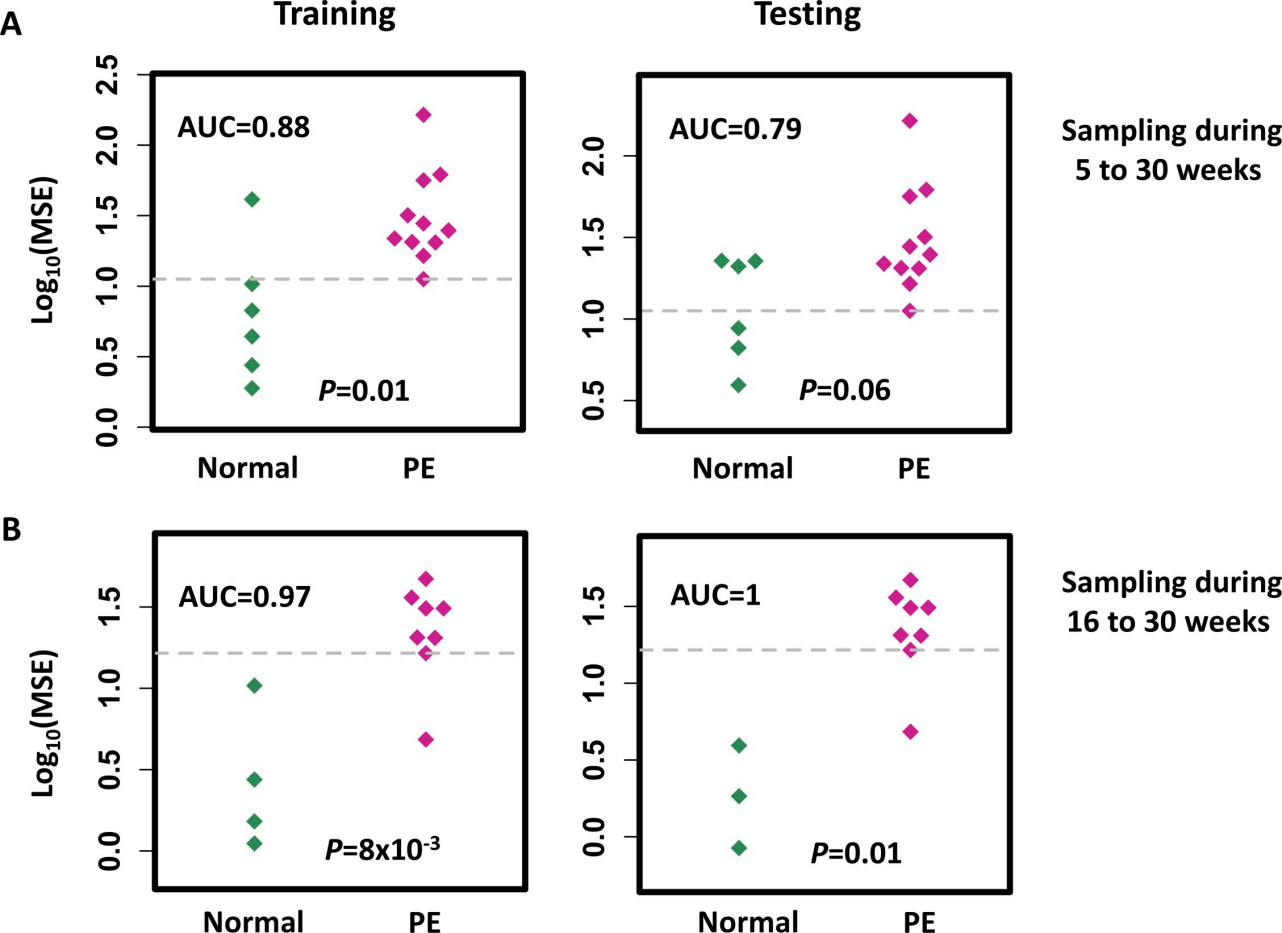
The mean squared error (MSE) of the EN model in estimating the GA of normal and PE patients in the training and testing cohorts, respectively. Mann-Whitney U-test P-value was calculated to measure the difference in MSE between the normal and PE patients. The cut-off point (grey dotted line) shows the maximum value of the sum square of the sensitivity and 1-specificity on classification of the training cohort of normal and PE women at blood sampling during (A) 5–30 and (B) 16–30 weeks of gestation.

Our placenta-related protein-based GA estimation characterized the gestational progression of normal term pregnancies. Significant random disruptions of this normal “term” model were observed in women with PE. Logarithm-transformed MSE of our EN estimations were utilized to define the binary classifications to identify risk for impending PE (Fig 4). For samples collected at 5–30 weeks’ GA, the MSE metric differentiated normal women from those who developed PE (Mann-Whitney U-test P = 0.01 on the training cohort, and P = 0.06 on the testing cohort) with areas under the curve (AUCs) of 0.88 on the training and 0.79 on the testing cohorts. An optimized cutoff value calculated on the training data yielded a positive predictive value (PPV) of 0.79 with a sensitivity of 1.00, and a negative predictive value (NPV) of 1.00 with a specificity of 0.50 on the testing data. In contrast, in the 16–30 weeks of gestation window, performance was improved: Mann-Whitney U-test (P = 8x10-3 on the training and P = 0.01 on the testing) and AUC of 0.97 on the training and 1 on the testing data, PPV of 1.00 with a sensitivity of 0.88, and NPV of 0.75 with a specificity of 1.00 on the testing data. These results may be more sensitive than using a single biomarker on the testing data in a window of 16–30 weeks of gestation (with AUCs of 0.53 for LEP; 0.76 for CHSL1; 0.58 for ELA; 0.53 for activin A; 0.65 for sFlt-1; and 0.65 for PlGF). Thus, our results demonstrate that significant disruptions in the protein-based GA estimation can be used to identify a woman’s risk for developing impending PE.

Our EN model was also further tested to see if the different PE subtypes could be identified [46, 47]. For samples collected at 5–30 weeks’ GA, the MSE metric had an AUC of 0.82 (0.90 for training and 0.73 for testing) and 0.85 (0.86 for training and 0.83 for testing) for differentiating normal women from those who developed early-onset PE or late-onset PE, respectively, and an AUC of 0.80 (0.86 for training and 0.74 for testing) and 0.90 (0.92 for training and 0.88 for testing) for differentiating normal women from those who developed mild or severe PE, respectively. For samples collected at 16–30 weeks’ GA, the MSE metric had an AUC of 0.95 (0.92 for training and 1 for testing) and 1 for differentiating normal women from those who developed early-onset or late-onset PE, respectively, and an AUC of 0.97 (0.95 for training and 1 for testing) and 1 for differentiating normal women from those who developed mild or severe PE, respectively. There was no significant differences (P > 0.05) in performance with regard to differentiating women who develop early- or late-onset PE, or those who develop mild or severe PE.

### A placenta-related, protein-based GA estimation with reduced number of features

Due to the lack of robustness of the mouse ELA ELISA assay, we tested the performance of our EN-based model excluding ELA. The model had an R2 of 0.72 and 0.61 on the training and testing cohorts, respectively. Disruptions in protein profiles were observed at < 30 weeks of gestation in women who developed PE (R2 = 0.27). Similar to the 6-protein model, the 5-protein model was still able to estimate GA during normal pregnancies.

### Comparative analysis of serological GA estimation between the human and a mouse model

**Fig 5:**
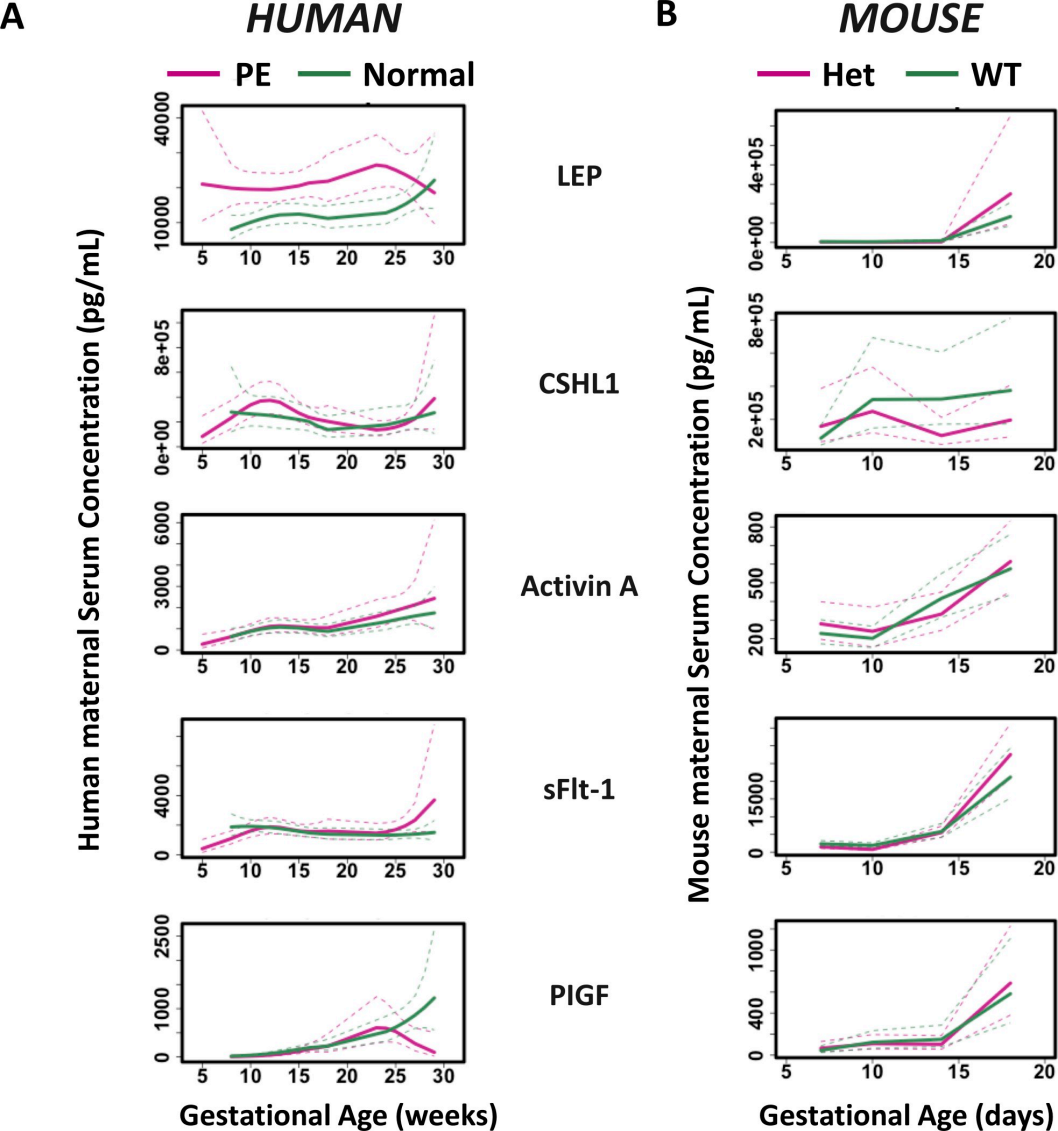
Loess smoothing of the serum levels of the 5 placenta-related proteins in (A) human and (B) mice as a function of GA for both normal and (A) PE or (B) HO-1 Het samples. The x axis represents GA, and the y axis represents serum concentrations. Red curves represent patterns of PE women and HO-1 Het mouse. Green curves represent patterns of normal pregnant women and WT mouse. Dotted lines represent 95% confidence intervals.

We hypothesized that similar temporal placenta-related protein expression patterns should be conserved in mouse pregnancies, therefore, we explored our EN-based 5-protein model to normal (n = 3 with 12 samples) and pregnant HO-1 Het mice (n = 4 with 15 samples), a mouse model reflecting PE-like symptoms. Model coefficients were adjusted to establish the link between GA (in days) and the targeted serum protein levels. The model had an R2 of 0.85 (P = 2 x 10–4) for pregnant WT, while the R2 was reduced to 0.30 (P = 0.07) for HO-1 Het mice with PE-like symptoms. Fold changes of protein levels of HO-1 Het over normal pregnancies were calculated and then compared between the human and mouse in early (human: 5–26 weeks; mouse: 7.5–14.5 days) and late (human: 27–38 weeks; mouse: 18.5 days) gestations separately. The largest fold change was observed in LEP at late gestation of mice (Fig 5). Unlike mice, LEP levels were elevated in women with PE in early gestation (Fig 3, S1 Fig, and Table 2). Fold changes of sFlt-1 and PlGF in mice increased from early to late gestation. The temporal patterns of sFlt-1 in mice were similar to those in human, which decreased in early gestation and increased in late gestation of complicated pregnancies (Fig 5). In contrast, PlGF significantly decreased in women with PE after 27 weeks’ GA, but not in HO-1 Het mice at late gestation.

## Discussion

The placenta plays a key role in fetal development, where cell communication occurs to support nutrition acquisition, immune adaption, and other functions of maternal-fetal interaction [48, 49]. Placental proteins are expressed in a time-dependent manner and cross-talk with other organs, such as the thyroid, pituitary, and ovary, and are necessary to ensure normal fetal development. Characterization of the temporal patterns of circulating placental proteins may serve as a basis for understanding the biology underlying both normal and pathological pregnancies. Our results support our hypothesis that multivariate modeling of the levels of circulating placental-secreted proteins, LEP, CSHL1, ELA, activin A, sFlt-1, and PlGF can be used to estimate GA during the course of a normal pregnancy, but not in women who develop PE. The longitudinal placental-related protein profile in sera was also observed in pregnant WT mice, but not in pregnant HO-1 Het mice.

Early diagnosis of PE remains a challenge in clinical settings. The traditional diagnosis of PE is based on the presence of maternal hypertension and proteinuria [50]. sFlt-1 and PlGF are well-established PE biomarkers [51] with clinical prognostic utility in the management of PE. The ratio of sFlt-1 and PlGF has been shown to effectively differentiate PE from normal term pregnancies, but only after 25 weeks of gestation [27]. Previous transcriptomic [52–58] and proteomic [2, 59–63] profiling of normal and complicated pregnancies have identified disease-specific expression patterns and signaling networks, which suggest candidate biomarkers for possible early clinical diagnoses and for offering new biological insights. Our findings suggest that a composite placental-related protein panel from serial blood collection (for MSE calculations) may provide a diagnostic test to assess PE earlier (~10 weeks of gestation) than previously suggested by sFlt-1 and PlGF (after 25 weeks of gestation as observed in this study). The diagnostic performance of the panel was similar among the different types of PE (early-onset vs. late-onset, mild vs. severe). Compared to a recent proteomics study where different models were predictive at different GA windows [64], our model with 6 proteins works across 5–30 wks of GA, which is more useful when exact gestational age is unknown. Therefore, this model may offer a new investigational approach towards the understanding of placental biology during pregnancy as well as guiding innovative methods for PE diagnosis.

Our findings of serum protein levels during a normal pregnancy are consistent with those from previous studies. They are in line with the ranges reported in healthy pregnancies and have similar patterns during the pregnancy as previous results [65–73]. We found that LEP increased continuously during the first and second trimesters. Activin A remained stable between 10–20 weeks of gestation and increased late in the second trimester. sFlt-1 levels were also unchanged before 30 weeks, while PlGF progressively rose over pregnancy. We further integrated the quantitative trending information of each individual protein into a continuous regression model that expressed GA as a linear combination of the levels of proteins.

An important finding of the study is that women who develop PE had serum LEP levels much higher than normal pregnant women in early gestation (<25 weeks). It is consistent with findings from other studies [74, 75], which indicated that LEP can serve as an early biomarker of PE. It is intriguing that the serum LEP levels increased weeks or months prior to the onset of PE (*P* = 3x10^-6^ at 15–25 weeks, while the onset dates of PE were 29 weeks or later in our study), suggesting that LEP itself may be involved in the early pathogenesis of the disease. Given that LEP is a master regulator of energy expenditure, the observations hint that placental insufficiency as a consequence of an energy imbalance may be a precursor to PE that is manifested as hypertension in mid to late gestation. Furthermore, since LEP is a placenta-related protein, its early increase observed in women who develop PE may support the hypothesis that early-onset PE is a placenta-based disease [46, 47]. LEP may be associated with placental vasculature defects, or even cause these changes.

Data from our HO-1 Het mouse studies suggest an association between placental HO-1 and the development of PE observed in humans [76] and provide a window of the mechanism of how a deficiency in HO-1 may affect the placental vascular network and leads to PE-like symptoms as mediated by increases in sFlt-1 [38]. Disruptions in placental vascular formation and spiral artery remodeling has been implicated as a causative factor of PE. Previous studies have shown that HO-1 expression is reduced in placentas of women with PE [76], and that HO-1 is involved in placental vascular network development [77]. The role of HO-1 in placental malfunction was further evaluated with HO-1 Het mice [38], revealing that a deficiency of maternal HO-1 results in an impaired placental vascular network and spiral artery malformation, and causes PE-like symptoms such as high diastolic blood pressures and elevated sFlt-1 levels [28].

Our HO-1 Het mouse study provided additional insights into the PE in terms of placental protein changes, especially relating to the role of LEP in its pathophysiology. Here, we observed that: serum LEP, CSHL1, Activin A, sFlt-1, and PlGF levels were highly correlated with GA in normal WT mice during pregnancy; In addition, this correlation was disrupted in pregnant HO-1 Het mice; Finally, both serum sFlt-1 and LEP levels were higher in pregnant HO-1 Het mice compared with normal pregnant WT mice. These findings were consistent with what we observed in human sera, and demonstrated that our EN model developed using human sera for identifying impending PE early in gestation could be duplicated in mice. Furthermore, the characterization of temporal pattern of LEP expression in mice may help us understand the pathophysiology of PE. The main action of LEP is in the maternal interface regulating angiogenesis, growth, and immunomodulation in the placenta during the early stages of pregnancy [78–84]. Although a dysregulation of LEP levels has been found to correlate with the pathogenesis of various pregnancy disorders [85], including PE, the exact mechanism of action of LEP and its upstream regulation remain unknown. Our characterization of serum placental protein profiles in the pregnant HO-1 Het mouse provides evidence of an association between HO-1 deficiency and an upregulation of LEP in this PE-like murine model. Taken together with the significant correlation of LEP levels at < 25 weeks’ GA and impending PE in our human pregnancy cohort may indicate a mechanistic role of LEP and HO-1 in the pathogenesis of PE, and lead to the identification of novel treatment strategies, such as pravastatin [86].

We also note that placenta-related proteins have distinct temporal patterns between human and rodent pregnancies. PlGF is significantly down-regulated after 27 weeks’ GA in humans with complicated pregnancies while the down-regulation was not observed in mice. For LEP, its maximum differentiating power is found at early gestation (< 25 weeks) for humans but not at early gestation in mice. The differences in placenta-related protein expression profiles between humans and mice may be explained by their differences in placental structures (e.g. a choriovitelline placenta is initially present in mice but absent in human; trophoblast cell invasion is restricted in mice but deep in human) and different placental endocrine functions [87–89].

This study has several limitations. First, the sample sizes for our human cohorts were small, and our population lacked racial heterogeneity. Second, the time intervals of blood collections between two serial samples varied (3–31 weeks for normal, and 3–25 weeks for PE). Most samples were collected in the first or second trimester. Only 12 normal and 9 PE patients had samples collected in the third trimester. Third, serum concentrations of LEP can be influenced by maternal status [90, 91]. We addressed this through the normalization to maternal BMI (S2 Fig) and found the temporal pattern in LEP remained. Fourth, variations in circulating protein levels could be due to the contributions from other tissues besides the placenta. Meta-analysis of PE and GA-matched uncomplicated pregnancy-associated placental gene expression patterns, including the targeted analytes of this study, has revealed similar expression trending along the gestations and differentiation between normal women and those who develop PE [92]. Fifth, ELA was not included in the rodent analyses due to the lack of the robustness of the mouse ELISA assay. Sixth, missing clinical information, such as intrauterine growth restriction, might limit our insights into the pathophysiology of PE. Future study with comprehensive clinical data plus placenta-related proteins like PlGF may give better results in PE assessment [93]. Finally, although the expression profiles of our candidate proteins may have clinical utility to assess impending PE, the robustness of this model can be greatly improved with more frequent blood collection times and a larger sample cohort.

## Conclusions

Longitudinal EN analysis of the circulating pregnancy-associated, placenta-related protein expression throughout pregnancy revealed patterns of the normal temporal progression of human gestation that can estimate GA. The elevated MSE of the EN metric, quantifying the disruptions of the estimation, offers a potential approach to identify impending PE. The protein markers in sera shared by the human and mouse and their significant associations with GA are conserved. In addition, PE-related patterns found in human are preserved in normal and HO-1 Het pregnant mice. This provides direct evidence of the causative action of HO-1 deficiency in the upregulation of LEP in a PE-like murine model and may reveal a new therapeutic target, such as HO-1, to prevent placental vascular disorders [86]. All of these demonstrate that the exploration of the temporal expression patterns of the placenta-related proteins in rodent models can be used to study the biology of human pregnancy disorders such as PE.

## Supporting information

S1 Fig Maternal serum concentrations of the 6 selected placenta-related proteins plotted by GA intervals during pregnancy. Mann-Whitney U-test *P*-values are shown.
(PDF)

S2 Fig Maternal serum concentrations of (A) LEP and (B) LEP normalized to body mass index (BMI) (pg/mL/kg/m^2^) shown as a function of GA in normal term (red line) and PE (green line) pregnancies. Loess smooth function was applied. Color-coded dotted lines: show the 90% confidence interval for each cohort.
(PDF)

S1 File EN modeling procedures. (PDF)

S1 Table Comparisons of the serum levels of each protein between normal and PE pregnancies. Signed differences in mean (PE minus normal; pg/mL) were calculated.
(PDF)

## Abbreviations

PE: Preeclampsia
CSHL1: Chorionic somatomammotropin hormone like 1
LEP: Leptin
GA: Gestational age
sFlt-1: Soluble fms-like tyrosine kinase
PlGF: Placental growth factor
HO-1: Heme oxygenase-1
ELA: Elabela
WT: Wild-type
EN: Elastic net
MSE: Mean squared error
AUC: Area under the curve
ROC: Receiver operating characteristic
PPV: Positive predictive value
NPV: Negative predictive value
BMI: Body mass index
