# Supplementary figures and images for: Changes in pregnancy-related serum biomarkers early in gestation are associated with later development of preeclampsia
Source: PLoS One. 2020 Mar 3;15(3):e0230000. doi: 10.1371/journal.pone.0230000 (PMC7053753; doi:10.1371/journal.pone.0230000)

S1 Fig.

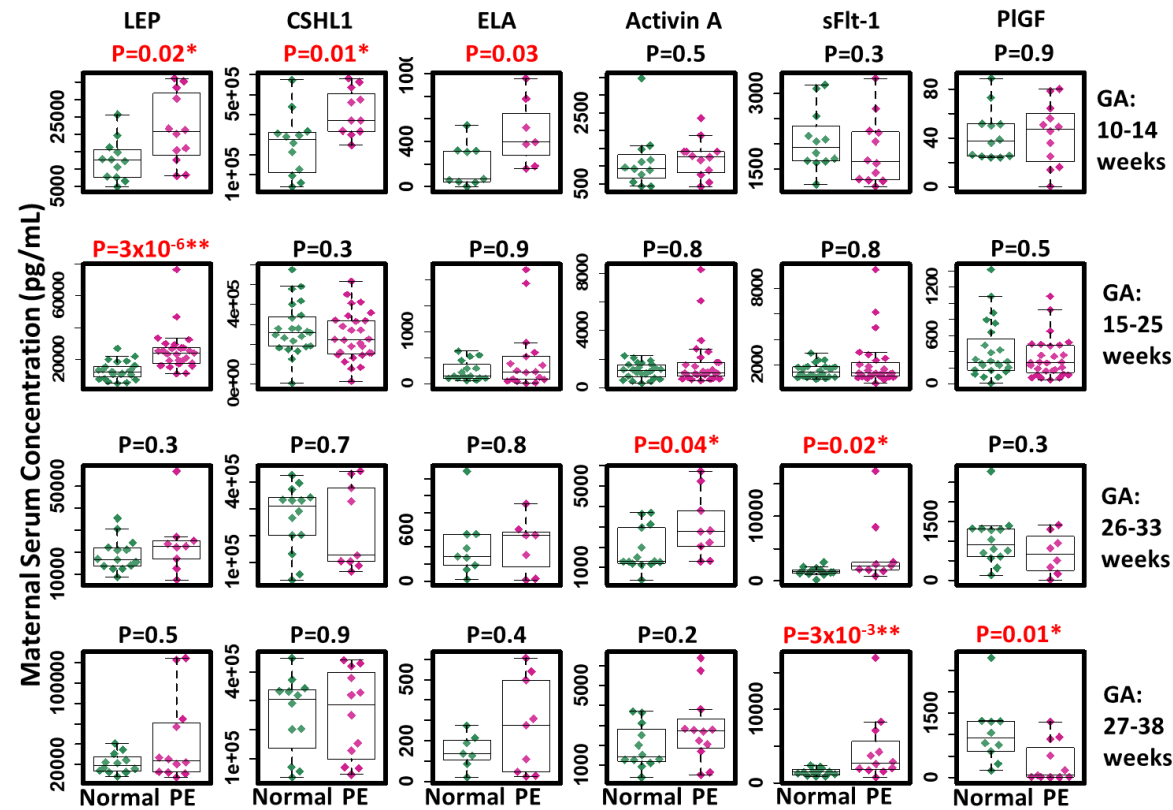

Supplement: S1 Fig — Mann-Whitney U-test P-values are shown. (PDF) [file pone.0230000.s001.pdf]

S2 Fig.

PE  
Normal

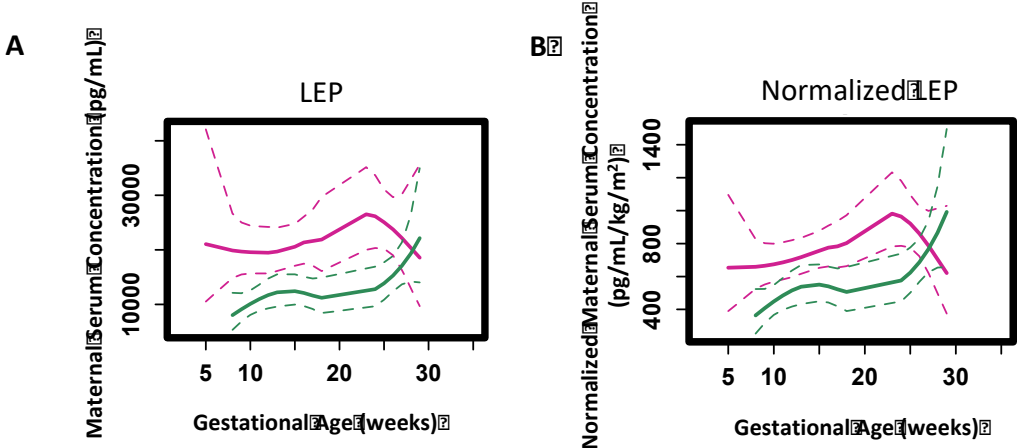

Supplement: S2 Fig — Maternal serum concentrations of (A) LEP and (B) LEP normalized to body mass index (BMI) (pg/mL/kg/m2) shown as a function of GA in normal term (red line) and PE (green line) pregnancies. Loess smooth function was applied. Color-coded dotted lines: show the 90% confidence interval for each cohort. (PDF) [file pone.0230000.s002.pdf]
