# Supplementary material for: Changes in pregnancy-related serum biomarkers early in gestation are associated with later development of preeclampsia
Source: PLoS One. 2020 Mar 3;15(3):e0230000. doi: 10.1371/journal.pone.0230000 (PMC7053753; doi:10.1371/journal.pone.0230000)
Supplement: S1 File — (PDF) [file pone.0230000.s003.pdf]

S1 Text.

A 10-fold cross-validated elastic net (EN) algorithm was used for multivariate modeling of the ELISA data. The model searches for an optimum β to minimize the least squared loss function with EN penalty:

$$L(\lambda, \beta) = |y - X\beta|^2 + \lambda[\frac{(1-\alpha)|\beta|^2}{2} + \alpha|\beta|] \quad (1)$$

where $X=(x_1,\ldots x_6)$ is a matrix of 6 analytes, with $x_j=(x_{1j},\ldots x_{nj})^T$, where $j=1,\ldots 6$. $y = (y_1,\ldots y_n)^T$ is the response (i.e., current GA). n is the number of samples in the training cohort. $|y - X\beta|^2$ is the squared loss function. $\lambda[\frac{(1-\alpha)|\beta|^2}{2} + \alpha|\beta|]$ is the well-known EN penalty used for controlling the model complexity. The parameters of each penalty were controlled by α and λ. α was set to 1 and λ was set to 0.208, which maximizes the predictive value of model measured by R^2 in the cross-validation (see Figure below). The model is thus reduced to a lasso-regularized regression.

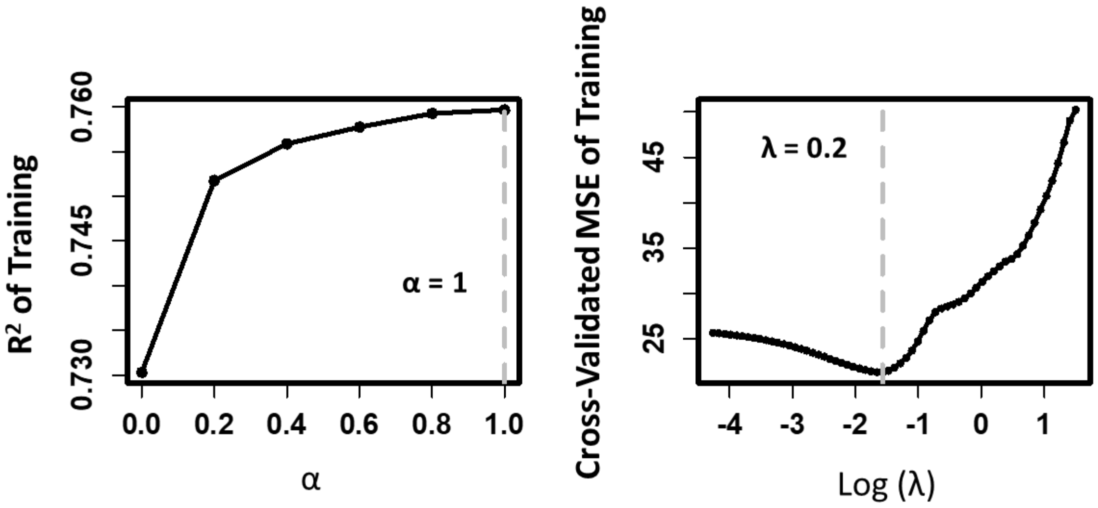

Figure. Performance of EN model with respect to α and λ in our training cohort. Left: R^2- value of the model with respect to α when λ was set to give the minimum cross-validation mean squared error (MSE). Right: Cross-validation MSE with respect to λ when α = 1.

The mean squared error (MSE) of the GA model was used to separate PE patients from women with normal pregnancies. Specifically, assuming a woman had estimated GA of $\hat{y}_{k1},\ldots,\hat{y}_{km}$ for samples collected at the observed GA of $y_{k1},\ldots y_{km}$, the MSE of the model for this woman is:

$$MSE = \frac{1}{m}\sum_{k=k1}^{km}(\hat{y}_k - y_k)^2 \qquad (2)$$

where m is the number of samples. To account for the randomness of errors, only women having 2 or more samples collected during pregnancy (i.e., $m \geq 2$) were included for the calculations.
