# Supplementary material for: Changes in pregnancy-related serum biomarkers early in gestation are associated with later development of preeclampsia
Source: PLoS One. 2020 Mar 3;15(3):e0230000. doi: 10.1371/journal.pone.0230000 (PMC7053753; doi:10.1371/journal.pone.0230000)
Supplement: S1 Table — Signed differences in mean (PE minus normal; pg/mL) were calculated. (PDF) [file pone.0230000.s004.pdf]

S1 Table.

	5–9 weeks' GA	10–14 weeks' GA	15–25 weeks' GA	26–33 weeks' GA	27–38 weeks' GA
**LEP**	15080	8858	12270	4935	21136
**CSHL1**	108141	161665	-37460	-35820	1964
**ELA**	-69	292	215	10	121
**Activin A**	-138	84	492	1175	946
**sFlt-1**	-645	-212	504	2831	2983
**PlGF**	20	0.1	-83	-337	-686
